# Inhibition of Intestinal Lipid Absorption by Cyanobacterial Strains in Zebrafish Larvae

**DOI:** 10.3390/md19030161

**Published:** 2021-03-18

**Authors:** Marta Bellver, Susana Lemos da Costa, Begoña Astrain Sanchez, Vitor Vasconcelos, Ralph Urbatzka

**Affiliations:** 1 Interdisciplinary Centre of Marine and Environmental Research (CIIMAR/CIMAR), University of Porto, Avenida General Norton de Matos, s/n, 4450-208 Matosinhos, Portugal; marbelcatala@gmail.com (M.B.); susanalemosdacosta@gmail.com (S.L.d.C.); bastrain@ciimar.up.pt (B.A.S.); vmvascon@fc.up.pt (V.V.); 2 Faculty of Science, Department of Biology, University of Porto, 4169-007 Porto, Portugal

**Keywords:** intestinal lipid uptake, fatty acids, zebrafish, obesity, feature-based molecular networking

## Abstract

Obesity is a complex metabolic disease, which is increasing worldwide. The reduction of dietary lipid intake is considered an interesting pathway to reduce fat absorption and to affect the chronic energy imbalance. In this study, zebrafish larvae were used to analyze effects of cyanobacteria on intestinal lipid absorption in vivo. In total, 263 fractions of a cyanobacterial library were screened for PED6 activity, a fluorescent reporter of intestinal lipases, and 11 fractions reduced PED6 activity > 30%. Toxicity was not observed for those fractions, considering mortality, malformations or digestive physiology (protease inhibition). Intestinal long-chain fatty acid uptake (C16) was reduced, but not short-chain fatty acid uptake (C5). Alteration of lipid classes by high-performance thin-layer chromatography (HPTLC) or lipid processing by fluorescent HPTLC was analyzed, and 2 fractions significantly reduced the whole-body triglyceride level. Bioactivity-guided feature-based molecular networking of LC-MS/MS data identified 14 significant bioactive mass peaks (*p* < 0.01, correlation > 0.95), which consisted of 3 known putative and 11 unknown compounds. All putatively identified compounds were known to be involved in lipid metabolism and obesity. Summarizing, some cyanobacterial strains repressed intestinal lipid absorption without any signs of toxicity and could be developed in the future as nutraceuticals to combat obesity.

## 1. Introduction

Obesity has nearly tripled since the 1970s and is considered a risk factor for the health of the global population [1]. It is predicted by 2030 that 28% of the global adult population will be obese and 38% overweight [2]. Of particular concern is the increasing percentage of childhood obesity, compromising their future health and putting a high pressure on health systems [3]. The complexity of this disease makes it necessary to approach diverse and complementary mitigation strategies. Although prevention through education and healthy lifestyle are long-term goals, many obese people will need additional strategies as drug treatments, medical devices and/or bariatric surgery [4,5]. The abnormal or excessive fat accumulation in obesity is associated with other diseases such as type 2 diabetes, hypertension, cardiovascular disease and several types of cancer [6,7]. It is characterized by a chronic positive energy imbalance, the digestion of dietary lipids by lipases being one of the main sources of fat absorption. In fact, gastric and pancreatic lipase inhibition is one of the targeted mechanisms to mitigate this disease [8]. Above the four long-term anti-obesity drugs approved by the Food and Drug Administration (FDA) and the European Medicines Agency (EMA) (liraglutide, naltrexone/bupropion combination, phentermine/topiramate extended release, and orlistat) [9,10], only orlistat targets this mechanism [8]. However, the invasive nature of medical interventions and the adverse secondary effects related to the approved anti-obesity drugs [9,11] make it necessary to find novel and safe compounds modulating lipid metabolism.

Natural products represent a great bioactivity potential since the chemical biodiversity of marine organisms is very wide and often underexplored [12,13]. Many marine natural products were elucidated from marine invertebrates, often in an endosymbiotic relationship with microorganisms [14,15]. Therefore, microbial-derived compounds are an important source of future natural drugs. Cyanobacteria constitute a diverse and widely distributed group of Gram-negative prokaryotes, found in freshwater, marine environments, and even terrestrial habitats [16]. They are well known to produce harmful toxins, but also a huge variety of secondary metabolites [16,17]. In fact, the cyanobacterial compound dolastatin 10 inspired the FDA-approved brentuximab vedotin for the treatment of soft carcinoma [18]. Dolastatin 15, obtained from *Lyngbya* sp., has also shown potential for the treatment of cancer [19]. Another compound isolated from cyanobacteria is Somocysteinamide A (*L. majuscula*/*Schizothrix sp.*/*Assemblage*), which has also been shown to be a possible drug due to its anticancer properties [20]. Regarding obesity, dietary supplementation with *Arthrospira* sp. has been related to anti-inflammatory effects and decreases in plasma triglyceride concentrations in humans, whereas *Nostoc commune var sphaeroides Kützing* diminished cholesterol uptake in mice [9]. Yoshinone A, isolated from *Leptolyngbya* sp., acts as a suppressant of the adipogenic differentiation in 3T3-L1 mice cells. This cyanobacterial compound is composed of a linear side chain and a γ-pyrone ring, in which its position is important to exhibit the inhibitory effect reported [[21]. The Blue Biotechnology and Ecotoxicology Culture Collection (LEGE-CC), located in the Interdisciplinary Center of Marine and Environmental Research (CIIMAR), comprises more than 1000 different cyanobacterial and microalgal strains (https://lege.ciimar.up.pt/, accessed 7 January 2021). A part of this collection has been already screened in our group for the identification of cyanobacterial strains with the potential for the treatment of obesity [4]. 13^2^-Hydroxy-pheophytin and other chlorophyll derivatives [22] were successfully isolated from marine cyanobacteria with lipid reducing activities.

The aim of this work was to identify strains of cyanobacteria that affect intestinal lipid uptake and processing, using zebrafish larvae as a relevant animal model. A PED6 fluorescent reporter was applied in a primary screening of cyanobacterial fractions for repression of intestinal lipases, and validated for normal functioning of the digestive physiology by analysis of intestinal protease activity. To decipher the potential effect of active fractions on various species of lipids, secondary assays were used to monitor the lipid uptake of fluorescent lipid analogues of short-chain fatty acids (SCFA) and long-chain fatty acids (LCFA). The alterations of lipid classes, as well as incorporation of LCFA into different lipid classes, was analyzed by thin-layer chromatography (TLC). Bioactive fractions were forwarded to metabolite profiling by LC-MS/MS and bioactivity-guided feature-based molecular networking to identify putative known or unknown compounds, which may be responsible for the observed bioactivities. This study demonstrates the potential of various cyanobacterial strains to modulate lipase activity and intestinal lipid uptake without any associated toxicity, which could be developed in the future to nutraceuticals for the combat of obesity.

## 2. Results

### 2.1. Primary Screening: Lipase Modulation

**Figure 1:**
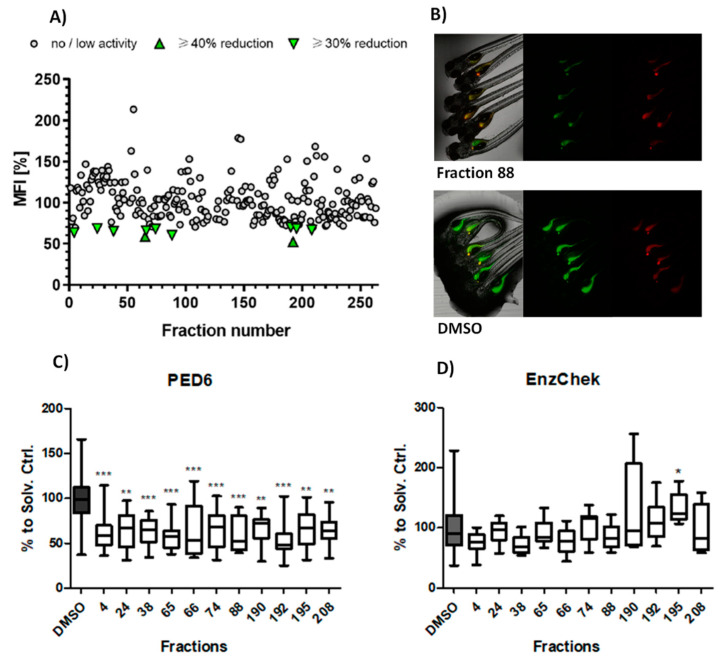
Lipase/protease inhibition activity. (**A**) Analysis of PED6 inhibition by the cyanobacterial library, shown as PED6-related mean fluorescence intensity (MFI). (**B**) Representative fluorescence images of an active fraction (88) and solvent control (DMSO). (**C**) Validation of the intestinal lipase inhibition of the 11 promising fractions; data are represented as box–whisker plots (5–95 percentiles), and significant differences are shown by asterisks (* = *p* < 0.05, ** = *p* < 0.01, *** = *p* < 0.001). (**D**) Absence of intestinal protease inhibition in all PED6 active fractions.

The aim of the primary screening assay was to identify fractions from cyanobacteria that could reduce intestinal PED6 reporter metabolism in comparison to the solvent control (dimethyl sulfoxide, DMSO). From a total 263 screened fractions, 11 (4.2%) reduced ≥ 30% the mean fluorescent intensity (MFI) related to PED6 cleavage. These active fractions were re-analyzed in an independent assay, and the data of both assays analyzed together for statistical differences. All bioactive fractions confirmed to significantly diminish MFI. These fractions belong to 7 cyanobacterial strains, 55% from freshwater and 45% from marine environments. Nine of these fractions (# 4, 24, 38, 66, 74, 88, 190, 195, 208) reduced MFI by ≥30% (moderate lipase inhibition effect), and 2 of these fractions (65, 192) diminished MFI by ≥40% (strong lipase inhibition effect) (Figure 1A–C).

The potential toxicity of the cyanobacterial fractions was monitored by three parameters: mortality of the larvae after 24 to 48 h, appearance of malformations, and reduction of the EnzChek reporter cleavage (intestinal protease activity). From the 263 screened fractions, 8 (3.04%) caused 100% mortality (# 49, 50, 51, 79, 80, 128, 152, 256), and 2 (0.8%) were related to a 50% mortality at the tested concentration (# 14, 89). Considering the 11 bioactive fractions, none showed signs of toxicity or interfered with intestinal protein digestion, with the exception of fraction 195, which significantly increased the protease activity (Figure 1D).

### 2.2. Secondary Screenings: SCFA and LCFA Uptake

**Figure 2:**
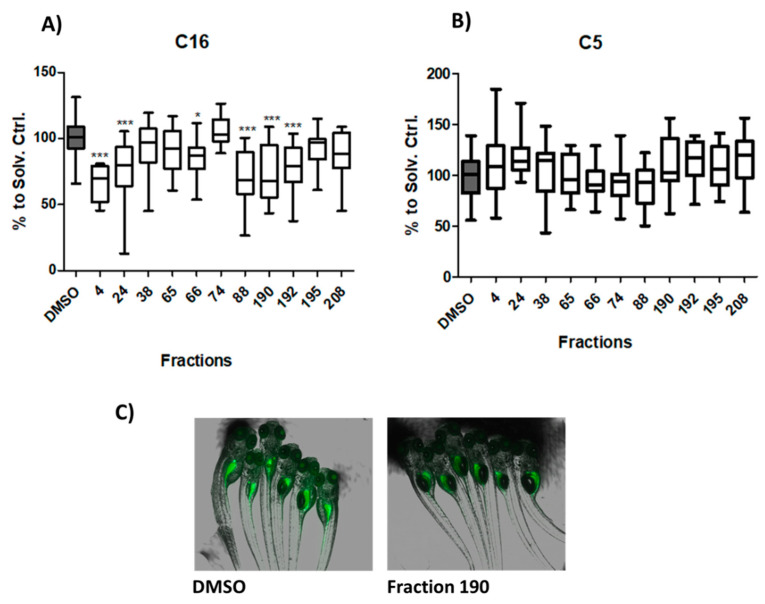
Long-chain fatty acid (LCFA) and short-chain fatty acid (SCFA) uptake modulation of promising cyanobacterial fractions. (**A**) Analysis of LCFA uptake, shown as BODIPY C16-related MFI reduction. (* = *p* < 0.05, *** = *p* < 0.001) (**B**) SCFA uptake modulation, shown as BODIPY C5-related MFI reduction. (**C**) Representative overlay images of brightfield and fluorescence microscopy of the solvent control (DMSO) and an active fraction (190).

Secondary assays were applied to analyze whether the selected bioactive fractions could influence the intestinal uptake of LCFA (BODIPY-C16) or SCFA (BODIPY-C5). Six fractions (# 4, 24, 66, 88, 190, 192) significantly reduced the uptake of BODIPY-C16 (Figure 2A,C). While five of these fractions showed moderate reduction effects, one of them (#4, freshwater strain) showed strong reduction by decreasing the MFI by ≥30%. No effect was observed on the intestinal uptake of BODIPY-C5 in comparison to DMSO (Figure 2B).

**Figure 3:**
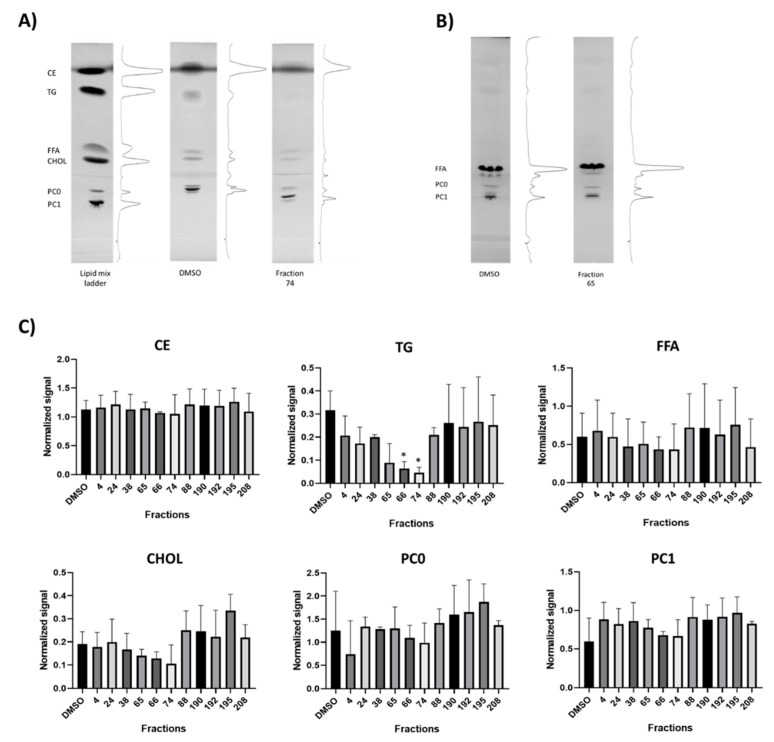
Alteration of lipid classes and lipid processing by promising cyanobacterial fractions. (**A**) Representative thin-layer chromatography (TLC) images showing the separation of different lipid classes isolated from zebrafish larvae (CE, TG, FFA, CHOL, PCO, and PC1), visualized by 0.01% primuline solution. Lipid ladder (mixed standards), solvent control (DMSO), and a representative active fraction (74) are shown. (**B**) Fluorescent TLC from zebrafish exposed to BODIPY-C16 before lipid isolation, showing the incorporation of C16 into different lipid classes. No significant differences were observed, as shown for a fraction with PED6 activity (65) compared to the solvent control (DMSO), and respective quantifications of all observed lipid classes in Figure S1. (**C**) Quantifications of lipid classes from TLC images (CE, TG, FFA, CHOL, PCO, and PC1) from bioactive fractions with PED6 inhibition. (* = *p* < 0.05).

Furthermore, the effects of the 11 selected bioactive cyanobacterial fractions were evaluated on the alteration of lipid classes or on lipid processing. Primuline staining of lipids on HPTLC plates demonstrated that 2 fractions (# 66, 74) significantly reduced the integrated density of triglycerides (TG) relative to solvent control, but other lipid classes were not affected (Figure 3A,C).

The incorporation and processing of LCFA into different classes of lipids was analyzed by fluorescent HPTLC using BODIPY-C16 (Figure 3B). However, any of the bioactive fractions significantly changed the incorporation of LCFA into lipid classes, but several fractions showed a tendency to diminish the lipid incorporation into free fatty acids (FFA) or phosphatidylcholine (Figure S1). The absence of significant effects on different lipid classes may be related to the fact that lipid extraction was done from whole embryos, and more localized effects are not detectable.

**Figure 4:**
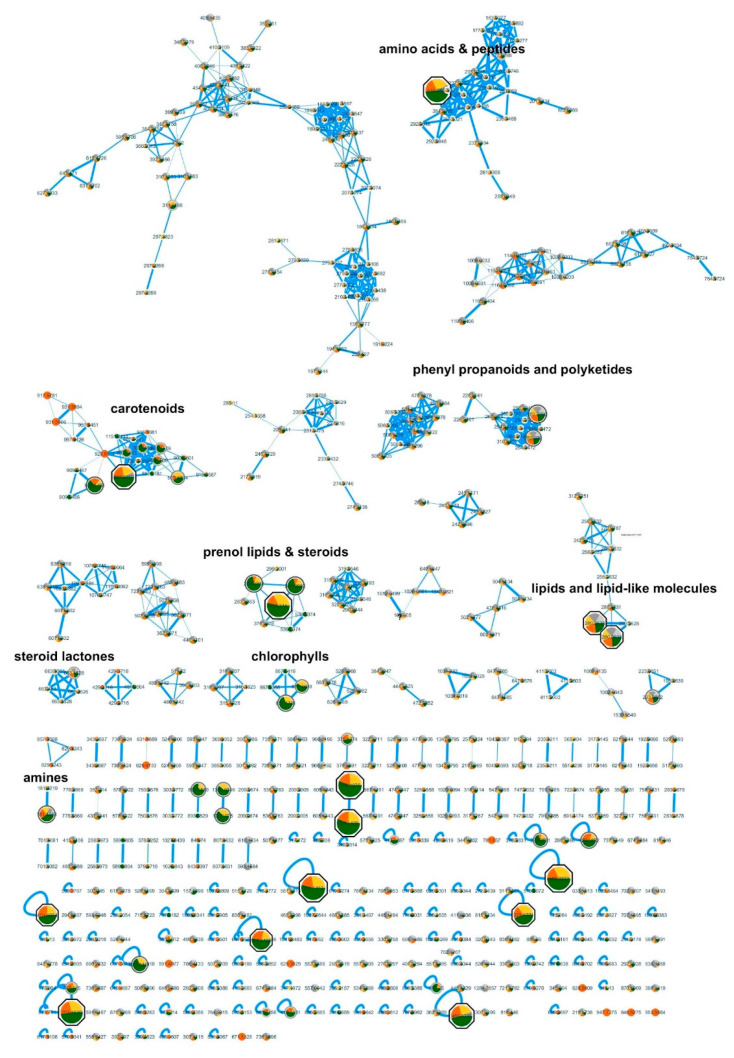
Bioactivity-guided molecular network using LC-MS/MS and bioactivity data. Bioactive mass peaks are highlighted by size, and the most significant are presented as octagonal shapes (*p* < 0.01 and correlation > 0.95). The color within the nodes corresponds to their relative quantity in the analyzed fractions: green, active fraction #192; yellow, non-active fraction #237; orange, non-active fraction #207; all from Synechoccocales.

For the bioactivity-based molecular networking of the LC-MS/MS data, the fraction with highest activity on inhibition of PED6 was chosen (192), together with two fractions without significant activity (207, 237) of the same type (A-fraction, extracted by hexane) and from the same cyanobacterial genera (Synechococcales). This resulted in the creation of a molecular network that allowed the comparison of quantities of present metabolites along active or non-active fractions. The most significant bioactive peaks were highlighted in the network by size (*p* < 0.01, correlation > 0.95) (Figure 4). The original Cytoscape file is provided in Supplementary Materials (Figure S2).

Bioactive peaks were manually checked in the Xcalibur software for peak intensity, H-isotopes, and Na+ adducts, and putative identifications searched in the Dictionary of Natural Products (DNP), Natural Products Atlas (NPA), and Global Natural Products Social Molecular Networking (GNPS). Contaminants were removed from the list, and finally 14 bioactive peaks were obtained representing known and putative novel compounds (Table 1). GNPS identifications are based on the similarity to MS2 fragmentation and shown in Table 1. Putative identifications from DNP and NPA are only based on MS1 data and were additionally compared to published MS2 data or, if not present, to theoretical fragmentations by ChemDraw software. As consequence, the following putative identifications were rejected and not further considered: M+H 203.1039, α-amino-2-carboxy-1-azetidinebutanoic acid by DNP (Δ 3.5 ppm); M+H 237.1096, 3,6-diamino-4,5-dihydroxyoctanedioic acid by DNP (Δ 3.9 ppm); M+H 361.1412, SF-2140 by NPA (Δ 3.5 ppm); M+H 417.314, 4,4′-diaponeurosporen-4-al by NPA (Δ −4.2 ppm); M+H 469.3285, acremolide C by DNP and NPA (Δ 1.6 ppm); and M+H 887.5669, 4-[2-O-11Z-octadecenoyl-β-glucopyranosyl]-4,4′-diapolycopene-4,4′-dioic acid by DNP and NPA (Δ −0.5 ppm). 

## 3. Discussion

In this study, zebrafish was used as a whole animal model for the screening of novel natural compounds from cyanobacteria that inhibit intestinal lipid uptake. The zebrafish (*Danio rerio*) has become a powerful model organism for research over the last 30 years [23]. The advantages of working with larval zebrafish are their external and rapid development, their capacity to produce numerous eggs (100–200 eggs/clutch), and their body transparency in their early days of development [24,25]. Concerning the study of metabolic diseases such as obesity, whole animal models have certain advantages, since in vitro studies cannot recreate metabolic processes in an organism and its complex interactions in a multi-organ context [26,27]. The optical clarity of zebrafish larvae is advantageous in the study of specific lipid digestive processes by using fluorescent stains, analogues, or reporters in vivo. The distribution of dietary lipids in zebrafish can be assessed by BODIPY–fatty acid and BODIPY–cholesterol analogues, and lipid processing with the fluorescent reporter PED6, a substrate, which is cleaved by phospholipase A2 (PLA2), releasing a fluorescent signal [28,29].

Dietary lipids are the most energetic substances ingested by mammals, and the modulation of its intake could be an efficient way for the treatment of obesity. Dietary lipids mainly consist of triacylglycerols (TAG), as well as cholesterol and phospholipids [30]. After partial hydrolysis in the stomach, these enter into the small intestine and get absorbed by enterocytes [28]. LCFA get incorporated through micelles into the enterocyte membrane, while free cholesterol and SCFA are uptaken via specific transporters [31] Once into the enterocyte, the fats bind to proteins to synthesize TAG again and form pre-chylomicrons in the endoplasmic reticulum. Then, pre-chylomicrons meld to form chylomicrons in the Golgi apparatus, where they are packed into vesicles [32] In this way, the chylomicrons (TAG-rich lipoproteins with a central body and a layer of phospholipids, cholesterol, and apoliproteins) access the lymphatic and blood stream [33]

In a previous study, a novel strategy was developed to analyze intestinal lipid uptake in zebrafish larvae in vivo. A primary screen with PED6 detected compounds causing defects on lipid processing, which was followed up by secondary assays for intestinal SCFA or LCFA uptake. In total, 50–70% of lipase inhibition was demonstrated for pure compounds, such as 1-methyl-3-[2-(methylamino)phenyl]quinoxalin-2-one and clofazimine [29]. Here, for the first time, the same strategy was applied on extracts/fractions from natural resources. In total, 11 out of 263 cyanobacterial fractions (4.2%) lowered the PED6 signal by 30–40%, which is either related to gastrointestinal absorption of phospholipids or to the inhibition of the intestinal phospholipase activity. The observed 30–40% inhibition of lipase activity can be considered as a promising result, since cyanobacterial fractions present a mixture of hundreds of compounds, which dilute the quantity of the bioactive ones. The 11 bioactive fractions were tested for defects in the uptake of SCFA and LCFA, or in the processing of fatty acids into lipids from various classes. Significant effects were observed for the uptake of C16 LCFA (fraction # 4, 24, 66, 88, 190, 192), which are highly present in refined oils and fats. Interestingly, no toxicity was observed for the bioactive fractions, which may enable the development of future nutraceuticals. Furthermore, a significant reduction of TG was observed for two fractions (# 66, 74). However, it is not clear whether a direct relationship can be drawn, since the fractions present a mixture of compounds, which could act on different targets and pathways, and since external feeding starts at later life stages, and thus, intestinal lipid absorption may not be involved in the observed reduction of TG. In concordance with this hypothesis, the screen of neutral lipid reduction by the Nile red fat metabolism assays in zebrafish larvae from the same fraction library resulted in the identification of bioactive fractions [4], which do not overlap with the observed fractions reducing intestinal lipid absorption. Future studies with the diet-induced obesity model in adult zebrafish should confirm whether the newly identified fractions with effects on intestinal lipid absorption cause a decrease in fat reservoirs in this model.

The Global Natural Products Social Networking (GNPS) site is an open-access knowledge base for mass spectrometry data [34]. This bioinformatic procedure is an interesting way of getting insights into the presence of unique compounds that differ between active and non-active fractions or extracts [35]. The further development of the methodology incorporating the correlation to bioactivity data resulted in the feature-based molecular networking (FBMN), which allows the profiling of the metabolites related to an observed bioactivity [36] The application of FBMN on our data from LC-MS/MS allowed the identification of 14 peaks (*p* < 0.01; correlation > 0.95), which could be responsible for the observed activity on lipid processing in zebrafish larvae (Figure S2, Table 1). Here, 11 peaks did not have a match in mass spec data bases (78%) and could be possibly novel compounds. All three peaks with a putative match in the databases had a link to obesity and lipid metabolism. Deoxycholic acid is a bile acid involved in the solubilization of dietary lipids in the intestine, and inhibited LCFA uptake in hepatocytes [ [37] A similar compound to match 1,2-dihydroxyheptadec-16-yn-4-yl acetate (2,4-dihydroxyheptadec-16-ynyl acetate) was described to inhibit a key enzyme of the fatty acid metabolism and is present in seagrass extracts [35]. 13^2^-Hydroxy-(13^2^S)-pheophytine a was isolated from a marine cyanobacterial strain in a bioassay-guided isolation procedure in the zebrafish larvae Nile red fat metabolism assay, and reduced neutral lipids at an EC_50_ concentration of 8.9 µM [21]. The target of PED6, the phospholipase A2 is known to produce arachidonic acid by hydrolysis of phospholipids, which is an important precursor molecule for inflammatory mediators such as prostaglandins, thromboxanes, and leukotrienes [38] Future work should analyze whether PED6 activity only relates to obesity and intestinal lipid uptake, or whether it is additionally related to inflammation. The strong bioactivity of selected fractions in vivo without toxicity makes them good candidates for the development of nutraceuticals or the future isolation of bioactive compounds.

## 4. Materials and Methods

### 4.1. Library of Fractions

A library of 263 fractions, extracted and fractionated from 46 strains of the Blue Biotechnology and Ecotoxicology Culture Collection (LEGE-Cc), was tested for lipase inhibition in the primary screening. The construction of this library is detailed in Costa et al. (2019), Supplementary Table S1 [4], including information regarding species names, strain codes from the collection, environment of isolation, sampling location, fractionation procedure, and sample codes.

### 4.2. Preparation of the Lipid Reporters

Lipid analogues and/or reporters were dissolved in DMSO to prepare stock solutions at the following concentrations: 5 mM (PED6, Invitrogen by Thermo Fisher Scientific, Waltham, MA, USA) and 6.4 mM (BODIPY-C5, BODIPY-C16; both Invitrogen by Thermo Fisher Scientific, Waltham, MA, USA). Following the manufacturer’s instructions, EnzChek (Invitrogen) was dissolved in 1× digestion buffer to prepare a 400 µg/mL stock solution. All the solutions were aliquoted in 20 µL volumes and stored at −20 °C.

### 4.3. Lipid Uptake Assays Using Zebrafish Larvae

According to the EC Directive 86/609/EEC for animal experiments, zebrafish larvae in non-independent feeding stages of development are not considered animal experimentation. Fish larvae were raised from 1 DPF to 5 DPF in egg water (60 mg/mL marine salt dissolved in distilled H_2_O) with 20 mM PTU (1-phenyl-2-thiourea, Acros Organics by Thermo Fisher Scientific, Waltham, USA) to inhibit melanogenesis. Primary lipase-protease screening assays and secondary assays for SCFA and LCFA uptake reduction screening were adapted from Clifton et al. (2010) [29]

Five DPF zebrafish larvae were transferred to 48-well plates at a density of 5–7 larvae/well (*n* = 5–7), and exposed for 24 h to cyanobacterial fractions (final concentration: 10 µg/mL). A solvent control (0.1% DMSO) was included in the assays. In lipase screening assays, the larvae were incubated for 6 h with PED6 at a final concentration of 0.5 µg/mL. Active fractions were validated in additional assays for effects on protease activity, being incubated for 6 h with EnzChek at a final concentration of 5 µg/mL, respectively. In secondary assays for SCFA or LCFA uptake, the larvae were incubated for 6 h either with BODIPY-C5 or BODIPY-C16 at a final concentration of 0.8 µM. Before imaging, the larvae were washed with egg water to minimize fluorescent background. The larvae were anesthetized in tricaine (MS-222, 0.03%, Sigma Aldrich by Merck, Darmstadt, Germany), and imaged in a fluorescence microscope (Leica DM6000) in the FITC channel (PED6, BODIPY-C5, and BODIPY-C16) or TRITC channel (EnzChek). Fluorescence imaging conditions were adjusted for control animals and then maintained for all treatment groups; the mean fluorescent intensity (MFI) was quantified in individual zebrafish larvae by ImageJ (http://rsb.info.nih.gov/ij/index.html, accessed 8 September 2020).

### 4.4. Lipid Extractions and Thin-Layer Cromatography

A fixed number of anesthetized larvae per well (*n* = 5) was transferred to 2 mL Eppendorf tubes. The samples were centrifuged for two minutes at 8000 rpm (VWR MicroStar 17R, Radnor, PA, USA), the supernatant discarded, and the tubes frozen at −80 °C. Lipid extractions were adapted from Folch (1957) and Flynn (2009) [39,40], and 500 µL of a mixture of 2:1 chloroform/methanol containing 0.01% butylated hydroxytoluene (BHT, Sigma Aldrich) was added to each sample, and homogenized using Ultra-Turrax (Ystral X1020). Then, 100 µL of 0.9% potassium chloride (KCl, Sigma Aldrich) was added and left on ice for 5 min, and vortexed and centrifuged at 2000 g for 30 min. Two liquid phases were observed: the lower layer was separated and dried. The samples were re-dissolved in 2:1 chloroform/methanol with BHT, and 10 µL of the samples or of the lipid mix (containing tripalmitin, palmitic acid, cholesterol, and L-α-phosphatidylcholine at 10 mg/mL, all Sigma Aldrich) was transferred to a HPTLC plate (nano silica glass plates, 10 × 10 cm, Sigma Aldrich). HPTLC plates were developed in a CAMAG flat-bottom chamber and dried. A solvent mixture of chloroform:methanol:water (12:6:1) was run twice up to 4 cm, and another of hexane:diethyl ether:acetic acid (160:40:3) was run once up to 9 cm. Visualization was done by spraying a 0.01% primuline solution (acetone:water, 6:4, Sigma Aldrich), and using a molecular imager Doc™ XR+ in SYBR Green (BioRad, Hercules, CA, USA). The quantification of integrated density (ID), which considers fluorescent intensity and peak area, was performed using ImageJ software. A similar approach was followed with fluorescently labeled zebrafish (incubated with fractions and BODIPY-C16). Here, the incorporation of BODIPY-C16 into different lipids was analyzed. Visualization was performed in the same imager in Alexa 488 settings.

### 4.5. LC-MS/MS

For liquid chromatography–high-resolution electrospray ionization tandem mass spectrometry (LC-HRESI-MS/MS, Thermo Fisher Scientific, Waltham, MA, USA), the fractions were dissolved in UPLC/MS grade acetonitrile at a concentration of 1 mg/mL, and 5 μL was injected per sample. Experimental conditions are described in detail in Ribeiro et al. (2020) [41].

### 4.6. Featured Based Molecular Networking

The raw data were converted to the mzML format using MSConverter and uploaded to MZmine2 [42]. The criteria for detection and quantification of spectral features were based on Duperron et al. (2020) [43]. The detection level was 1.0E6 for MS1 and 1.0E01 for MS2. The ADAP chromatogram builder was chosen with a minimum group size of 2, group intensity threshold and minimum high intensity of 1.0E06, and m/z tolerance of 0.005 or 10 ppm. Chromatogram deconvolution was done with ADAP wavelets, a signal-to-noise ratio of 10, minimum feature high of 1000, coefficient area threshold of 100, a peak duration of 0.02–1.0 min, and RT wavelength range of 0.02–0.6 min. The m/z range for MS2 pairing was 0.005 m/z or 10 ppm and an RT of 0.1. The isotope peak grouper had an absolute RT of 0.3 min, and maximum charge of 1, and the most intensive was chosen as representative isotope. Settings for the RANSAC algorithm of Joint Aligner had iterations 0, minimum number of points 80%, threshold 0.3, and same charge state. The peak finder was used for gap filling with an m/z tolerance of 0.005 or 10 ppm and RT of 0.1. For the peak list row filter, only peaks with MS2 scans were kept and peak numbering reset. As described in the bioactivity-guided molecular networking, bioactivity data were joined in the feature table and correlations run in the Jupyter notebook [36]

A molecular network was created with the feature-based molecular networking (FBMN) workflow [36] on GNPS (https://gnps.ucsd.edu, accessed 12 October 2020 [34]). The data were filtered by removing all MS/MS fragment ions within +/− 17 Da of the precursor m/z. MS/MS spectra were window-filtered by choosing only the top 6 fragment ions in the +/− 50 Da window throughout the spectrum. The precursor ion mass tolerance was set to 0.02 Da, and the MS/MS fragment ion tolerance to 0.02 Da. A molecular network was then created where edges were filtered to have a cosine score above 0.7 and more than 6 matched peaks. Then, the edges between two nodes were kept in the network if and only if each of the nodes appeared in each other’s respective top 10 most similar nodes. Finally, the maximum size of a molecular family was set to 100, and the lowest scoring edges were removed from molecular families until the molecular family size was below this threshold. The analogue search mode was used by searching against MS/MS spectra with a maximum difference of 100.0 in the precursor ion value. The GNPS library spectra were filtered in the same manner as the input data. All matches kept between network spectra, and the library spectra were required to have a score above 0.7 and at least 6 matched peaks. The molecular networks were visualized using Cytoscape software [44] The molecular networking job can be publicly accessed at https://gnps.ucsd.edu/ProteoSAFe/status.jsp?task=823f4c148e8a4cacafa8160a3d4118fe.

The output table of the Jupyter notebook was imported into the network, and nodes with significant bioactivity highlighted by size (*p* < 0.01, correlation >0.95). Additionally, neutral masses were searched in the Dictionary of Natural Products (http://dnp.chemnetbase.com/, accessed 11/01/2021), applying a search filter of +/− 0.001 m/z. The putative identifications, chemical formula, and mass differences in ppm are reported.

### 4.7. Statistical Analysis

Graphpad Prism (Version 8, San Diego, CA, USA) was used to generate graphs and to develop statistical analyses. Datasets were analyzed for normality using Shaphiro–Wilk or Kolmogorov–Smirnov normality tests, and for equal variances using Barthletts test. If the data passed assumptions for ANOVA, one-way ANOVAs with Dunnet’s post hoc tests (*p* value < 0.05) were performed to find the significant different fractions compared to the solvent control group. If the data did not meet those criteria, Kruskal–Wallis with Dunn’s post hoc tests (*p* value < 0.05) was applied.

## 5. Conclusions

Several of the screened cyanobacterial fractions had potent effects on intestinal lipid absorption in zebrafish larvae in vivo. These fractions inhibited intestinal phospholipase and LCFA lipid uptake, but did not have any toxic effects. The bioactivity-guided feature-based molecular networking approach identified 14 mass peaks, which could be responsible for the observed bioactivities, consisting of putative known and unknown compounds.

## Figures and Tables

**Table 1:** Putative identification of bioactive mass peaks by the Global Natural Products Social Molecular Networking (GNPS), Dictionary of Natural Products (DNP), and Natural Products Atlas (NPA). The table shows the 14 bioactive mass peaks that were obtained by bioactivity-guided networking filtered with the criteria of *p* < 0.01 and correlation > 0.95. GNPS identifications are based on the MS2 fragmentation, while the search criterion for DNP and NPA was the accurate mass range of m/z values +/− 0.001. For DNP and NPA identifications, MS2 fragmentation was confirmed by manual comparison to published MS2 data or theoretical fragmentation by ChemDraw. M, accurate mass; M+H, mass + hydrogen; RT, retention time; ppm, parts per million.

M+H	RT	Putative Identification	Adduct	Shared Peaks	MQ Score	ppm	Formula	Source
203.1039	0.8194							
237.1096	0.7999							
280.2628	19.0403	1,2-Dihydroxyheptadec-16-yn-4-yl acetate	[M+K]+	8	0.900225			GNPS
329.1721	3.5673							
337.104	1.87							
355.1875	4.411							
361.1412	1.3992							
371.1829	2.0569							
401.1934	1.154							
417.314	9.5136	Deoxycholic acid	[M+Na]+	9	0.81458			GNPS
469.3285	9.3014							
489.3332	9.3193							
887.5669	14.0394	13^2^-Hydroxy-(13^2^-S)-pheophytine A				−2.0	C55H74N4O6	NPA
913.8189	12.6052							

## Data Availability

All data are contained within the article and Supplementary Materials.

## Supplementary Materials

The following are available online at https://www.mdpi.com/1660-3397/19/3/161/s1: Figure S1: Quantifications of fluorescent HPTLC for incorporation of C16 into different lipid classes. Figure S2: Cytoscape file from bioactivity-guided molecular networking using LC-MS/MS and bioactivity data.
