# Supplementary material for: Inhibition of Intestinal Lipid Absorption by Cyanobacterial Strains in Zebrafish Larvae
Source: Mar Drugs. 2021 Mar 18;19(3):161. doi: 10.3390/md19030161 (PMC8003170; doi:10.3390/md19030161)
Supplement: Supplementary file 1 [file marinedrugs-19-00161-s001.zip › marinedrugs-1141366-SI/Supplementary Figure 1.pdf]

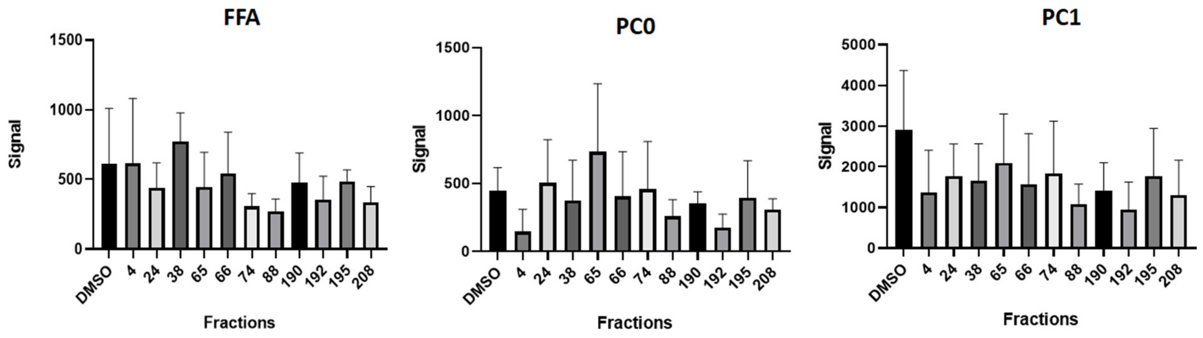

Supplementary Figure S1: Quantification of the incorporation and processing of LCFA into different classes of lipids of cyanobacterial fractions by ImageJ from fluorescent HPTLC plates.
